# One Nanoscale Zn(II)-Nd(III) Complex With Schiff Base Ligand: NIR Luminescent Sensing of Anions and Nitro Explosives

**DOI:** 10.3389/fchem.2020.536907

**Published:** 2020-10-14

**Authors:** Xia Liu, Xiaoping Yang, Yanan Ma, Jieni Liu, Dongliang Shi, Mengyu Niu, Desmond Schipper

**Affiliations:** ^1^College of Chemistry and Materials Engineering, Wenzhou University, Wenzhou, China; ^2^Department of Chemistry and Biochemistry, The University of Texas at Austin, Austin, TX, United States

**Keywords:** lanthanide complex, Schiff base ligand, nanoscale structure, NIR luminescence, luminescent sensing

## Abstract

One Zn-Nd complex [Zn_2_Nd_4_L_2_(OAc)_10_(OH)_2_(CH_3_OH)_2_] (**1**) was synthesized from Schiff base ligand bis(3-methoxysalicylidene)ethylene-1,2-phenylenediamine (H_2_L). **1** shows nanoscale rectangular structure with sizes of about 0.8 × 1.1 × 2.8 nm. **1** exhibits typical near-infrared luminescence of Nd(III) under the excitation of UV-visible light. Further study shows that the complex displays luminescent response behavior to anions and nitro explosives, especially with high sensitivity to H_2_${\text{PO}}_{2^{-}}$ and 2,4,6-trinitrophenol.

## Introduction

Construction of heterometallic d-f nanoclusters has received much interest during recent years because of their unique chemical properties (Peng et al., 2012; Wang et al., 2013; Yang et al., 2014; Andruh, 2015; Wen et al., 2019). Fluorescent response to ions and small molecules has received great attention because of the potential application in many areas such as medicine, biology, and environment (Jankolovits et al., 2011; Sun et al., 2015; Qi et al., 2017). As we know, luminescent lanthanide complexes can show emissions in both visible and near-infrared (NIR) ranges (900–1,600 nm) with sharp emission bands, large Stokes shifts, and long lifetimes (Hu et al., 2017; Ning et al., 2018). At present, many visible luminescent complexes with Tb(III) and Eu(III) ions have been used to detect analytes (Guo et al., 2011; Liu et al., 2013; Shi et al., 2015). However, compared with NIR fluorescent probes based on organic fluorophores (Yuan et al., 2013; Guo et al., 2014), very few NIR luminescent lanthanide complexes with Yb(III), Nd(III), and Er(III) ions have been reported to be used as sensors for the detection (Shi et al., 2019).

**Scheme 1:**
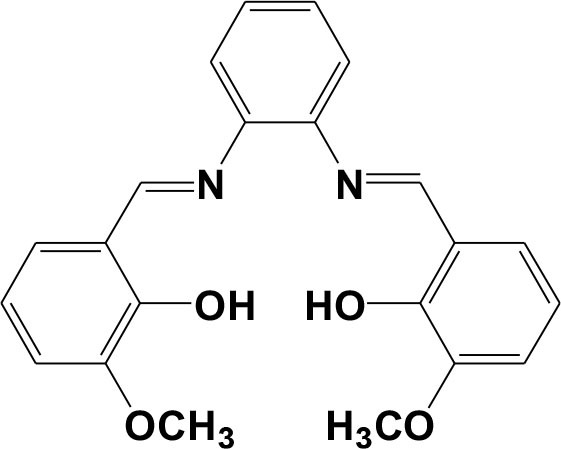
Schiff base ligand H_2_L.

Phosphates play a key role in biological energy storage and signal transduction, and nitro explosives such as 2,4,6-trinitrophenol (TNP) are very common ingredients of industrial explosives. Thus, many efforts have been made to design fluorescent sensors for phosphates (Yang et al., 2015; Sedaghat et al., 2019) and nitro explosives (Nagarkar et al., 2014; Liu et al., 2017). Our current research interests are in the design of lanthanide-based complexes with luminescent response to various ions and explosives (Jiang et al., 2018; Shi et al., 2019; Liu et al., 2020). Thus, we report here the synthesis and NIR luminescence properties of a Zn-Nd complex [Zn_2_Nd_4_L_2_(OAc)_10_(OH)_2_(CH_3_OH)_2_] (**1**) with Schiff base ligand bis(3-methoxysalicylidene)ethylene-1,2-phenylenediamine (H_2_L, Scheme 1). **1** has nanoscale rectangular structure with diameters of 0.8 × 1.1 × 2.8 nm. The complex shows interesting NIR luminescent response behavior to anions and explosives, especially to H_2_${\text{PO}}_{2^{-}}$ and 2,4,6-trinitrophenol (TNP) at ppm level.

## Experimental Section

### Preparation of [Zn_2_Nd_4_L_2_(OAc)_10_(OH)_2_(CH_3_OH)_2_] (1)

Zn(OAc)_2_·2H_2_O (0.30 mmol, 0.0658 g), NdCl_3_·6H_2_O (0.60mmol, 0.2154 g), and H_2_L (0.30 mmol, 0.0324 g) were dissolved in 50 mL MeOH at room temperature, and a solution of triethylamine in EtOH (1.0 mol/L, 1 mL) was then added. The mixture was stirred and heated under reflux for 30 min and then filtered. The yellow crystalline product of **1** was obtained by the slow diffusion of diethyl ether into the filtrate at room temperature after 1 month. The crystalline product was collected by filtering and then dried at 120°C in the oven for 2 h. Yield: 0.0981 g (25 %). m. p. > 200°C (dec.). Elemental analysis: found: C, 32.91; H, 4.12; N, 2.50%. Calc. for C_72_H_104_Zn_4_Nd_4_N_4_O_40_Cl_4_ ([Zn_2_Nd_4_L_2_(OAc)_10_(OH)_2_(CH_3_OH)_2_]·[ZnCl_2_(H_2_O)CH_3_OH)]): C, 32.68; H, 3.96; N, 2.12%. IR (CH_3_CN, cm^−1^): 1,703 (s), 1,551 (s), 1,484 (s), 1,289 (s), 1,239 (s), 1,193 (s), 1,072 (m), 963 (s), and 845 (s).

### X-Ray Crystallography

A Smart APEX CCD diffractometer was used to collect the X-ray data of **1** at 190 K. The structure was solved by the direct method (SHELX 97 program) (Sheldrick, 1997). Non-hydrogen atoms were refined anisotropically. Hydrogen atoms were included in the structure factor calculation but not refined. See http://www.rsc.org/suppdata/cc/ for the crystallographic data of **1** in CIF format (CCDC no. 1971956). The selected bond lengths and angles for the structure of **1** are listed in Supplementary Table 1.

For **1**: C_72_H_104_Zn_4_Nd_4_N_4_O_40_Cl_4_, triclinic, space group P-1, *a* = 9.6222(19), *b* = 14.771(3), *c* = 18.303(4) Å, α = 104.25(3)°, β = 98.18(3)°, γ = 103.42(3)°, *V* = 2,397.0(8) Å^3^, *Z* = 1, *Dc* = 1.833 g cm^−3^, μ(Mo-Kα) = 3.305 mm^−1^, *F*(000) = 1,312, *T* = 190 K. *R*_1_ = 0.0731, *wR*_2_ = 0.1934, GOF = 1.036.

## Results and Discussion

### Synthesis and Crystal Structure of the Complex

**Figure 1:**
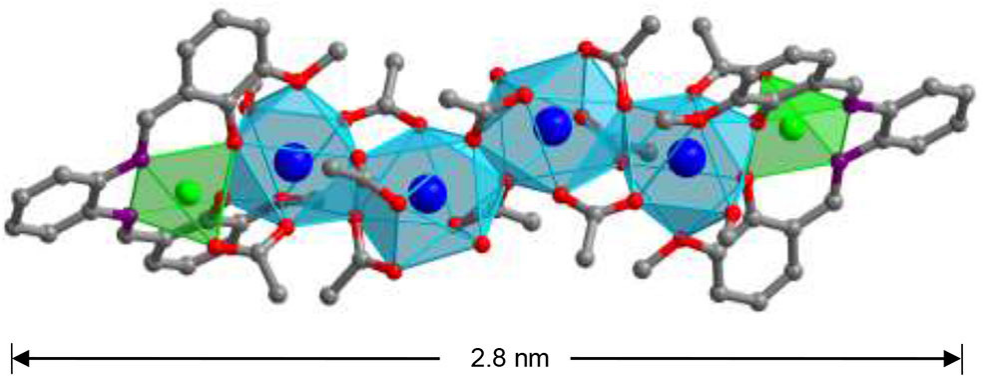
The X-ray crystal structure of **1** (Nd^3+^: blue, Zn^2+^: green).

The Schiff-base ligand H_2_L was prepared according to the literature report (Lam et al., 1996; Liu et al., 2020). For the synthesis of d-f complexes, the proportion of raw materials in the reaction may affect the composition of the product. Reaction of H_2_L with Zn(OAc)_2_·2H_2_O and NdCl_3_·6H_2_O in a molar ratio of 1:1:2 gave **1**, in which the ratio of L^2−^:Zn^2+^:Nd^3+^ is 1:1:2. The slow diffusion of diethyl ether into the reaction solution led to the formation of yellow crystalline product of **1**. This diffusion way helps to produce pure product of **1**, but results in the low yield (25%), because there are still a large number of product in the mother solution. In the crystalline solid product, zinc chloride ([ZnCl_2_(H_2_O)CH_3_OH)]) coexists with the complex. The crystal structure of **1** is shown in Figure 1. It is centrosymmetric with two equivalent ZnNd_2_L moieties linked by two OAc^−^ anions. The molecular dimensions of **1** are about 0.8 × 1.1 × 2.8 nm. In the ZnNd_2_L moiety, Zn^2+^ ion exhibits a square pyramidal geometry, coordinated with the O_2_N_2_ cavity of L^2−^. The coordination number of Nd^3+^ ions is nine, with a single cap square antiprism geometry. The L^2−^ ligand is coordinated with metal ions using two nitrogen and two oxygen atoms. In **1**, the average distance between neighboring Nd^3+^ ions is 4.088 Å. The bond lengths of Zn-N, Zn-O, and Nd-O are 2.036–2.047, 1.994–2.012, and 2.314–2.679 Å, respectively.

Because of the volatilization of solvent molecules in the product, the complex loses about 5% of the weight when heated before 100°C (Supplementary Figure 2, thermogravimetric analysis). It is stable until the heating temperature is about 200°C. Molar conductivity study indicates that **1** is neutral in CH_3_CN, in agreement with its crystal structure. This suggests that **1** remains its unique molecular structure in solution.

### Photophysical Properties and Response to Analytes

**Figure 2:**
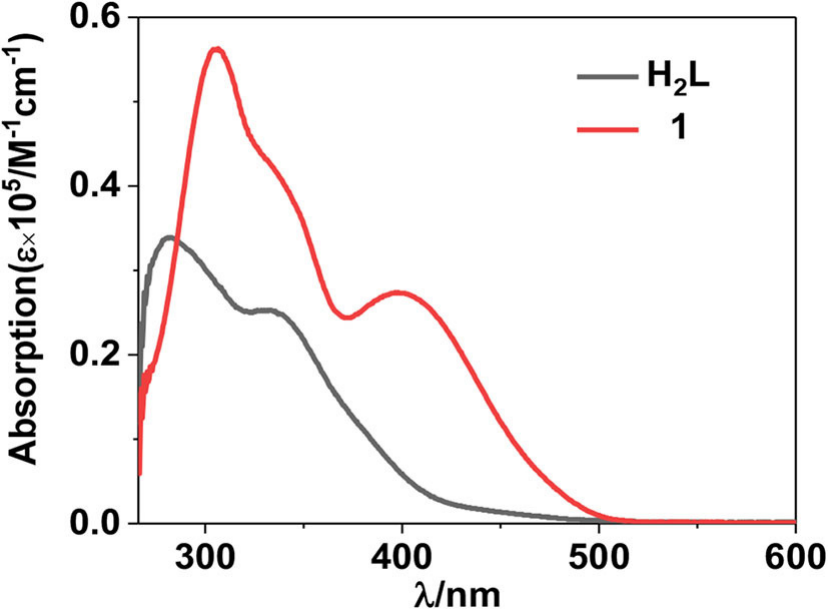
UV-visible spectra of the free ligand H_2_L and complex **1** at 298 K.

**Figure 3:**
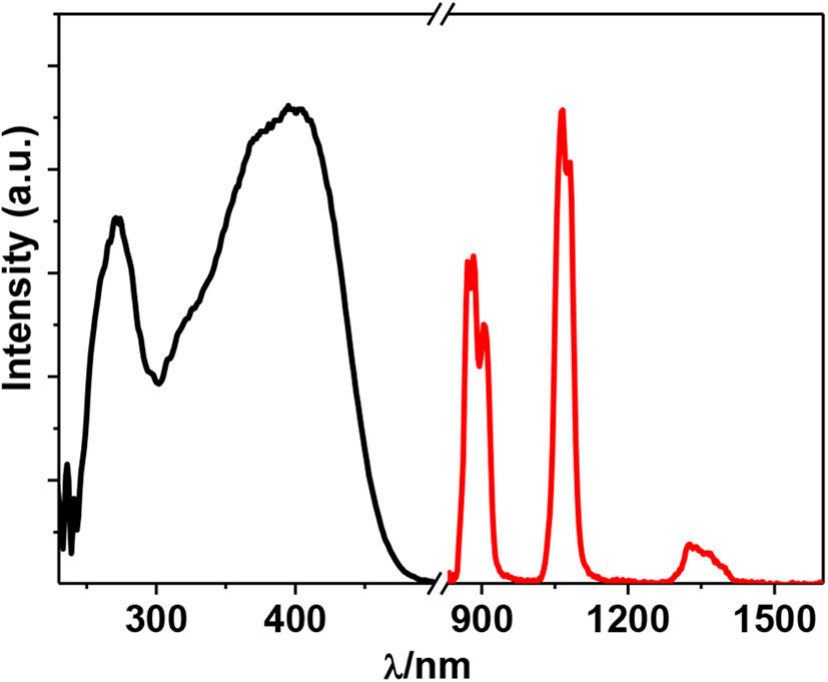
Excitation (λ_em_ = 1,054 nm) and emission (λ_ex_ = 395 nm) spectra of **1** (50 μM) in CH_3_CN at 298 K.

The free H_2_L shows UV-visible (UV-vis) absorption bands originated from the π − π^*^ transition, which are red-shifted in **1** due to the perturbation of metal ions to the transition (Figure 2). Metal organometallic chromophores with Zn(II) ion in **1** may efficiently transfer energy to lanthanide ions and sensitize lanthanide luminescence (“antenna effect”) (Xu et al., 2010). Thus, excited by ligand-centered absorption bands, **1** shows typical NIR luminescence of Nd^3+^ (${\;}^{4}{\text{F}}_{3/2}\to^{4}{\text{I}}_{\text{j}/2}$ transitions, *j* = 9, 11, and 13), and the most intense line is at 1,054 nm (${\;}^{4}{\text{F}}_{3/2}\to^{4}{\text{I}}_{11/2}$) (Figure 3). The complex exhibits broad ligand-centered excitation bands, indicating the ligand-to-metal energy transfer (LMET) in **1**. The NIR emission lifetime (τ) and quantum yield (Φ_em_) of **1** in CH_3_CN are found to be 6.2 μs and 0.8%, respectively.

**Figure 4:**
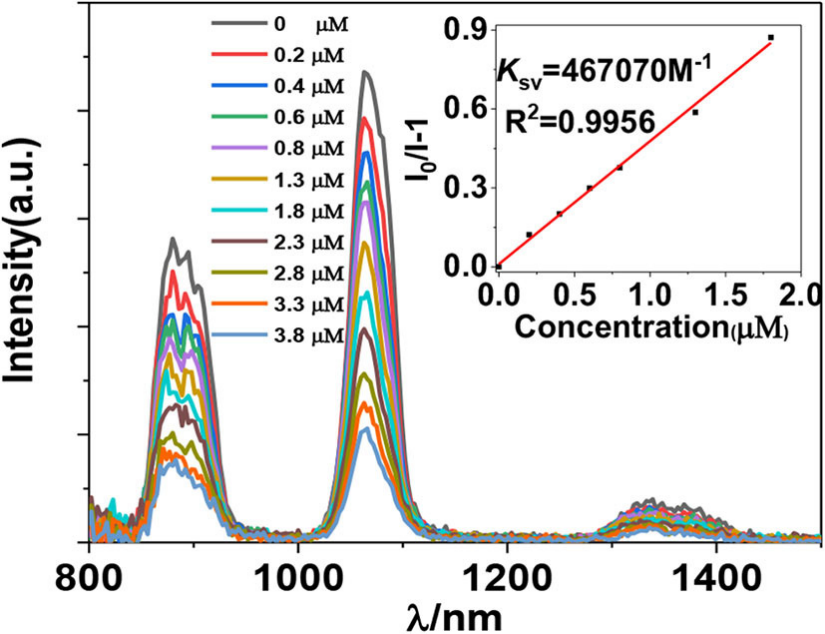
Lanthanide luminescent response of **1** (5 μM) to H_2_${\text{PO}}_{2^{-}}$ anion in CH_3_CN (λ_ex_ = 395 nm).

**Figure 5:**
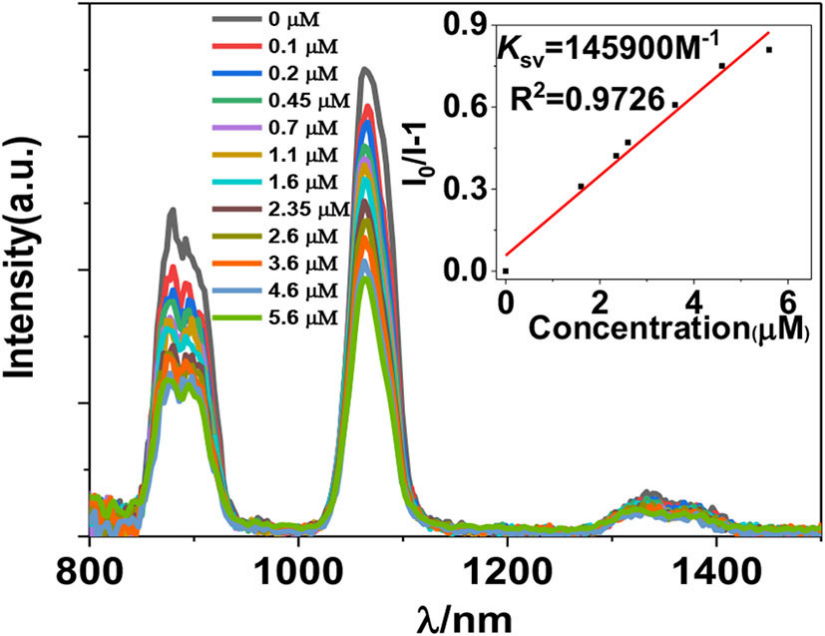
Lanthanide luminescent response of **1** (5 μM) to TNP in CH_3_CN (λ_ex_ = 395 nm).

The luminescent response of **1** toward anions H_2_${\text{PO}}_{2^{-}}$, F^−^, CN^−^, OH^−^, ${\text{SO}}_{4}{\;}^{2-}$, OAc^−^, Cl^−^, Br^−^, ${\text{CrO}}_{4^{-}}$, and ${\text{PF}}_{6^{-}}$, and nitro explosives 2,4,6-trinitrophenol (TNP), 4-nitrochlorobenzene (4-NBC), 2-nitrophenol (2-NP), nitrobenzene (NB), 4-nitrobenzaldehyde (4-NBAP), 4-nitrotoluene (4-NT), 1,3-dinitrobenzene (1,3-DNB), and 4-nitrobenzyl chloride (4-NCB) (Supplementary Scheme 1) has been studied in CH_3_CN. It was found that the addition of all anions and explosives results in a quenching of the lanthanide luminescence (Supplementary Figures 4, 5). It is noticeable that the addition of H_2_${\text{PO}}_{2^{-}}$ and TNP causes a more rapid decrease of the luminescence intensities than the addition of other anions and explosives (Figures 4, 5). For example, the emission intensities at 1,054 nm of **1** are decreased more than 50% when the concentrations of added H_2_${\text{PO}}_{2^{-}}$ and TNP are 1.8 and 5.6 μM, respectively, which are much lower than those of other anions and explosives (Supplementary Figures 4, 5). These results indicate that **1** shows high selectivity to H_2_${\text{PO}}_{2^{-}}$ and TNP through lanthanide luminescent response.

**Figure 6:**
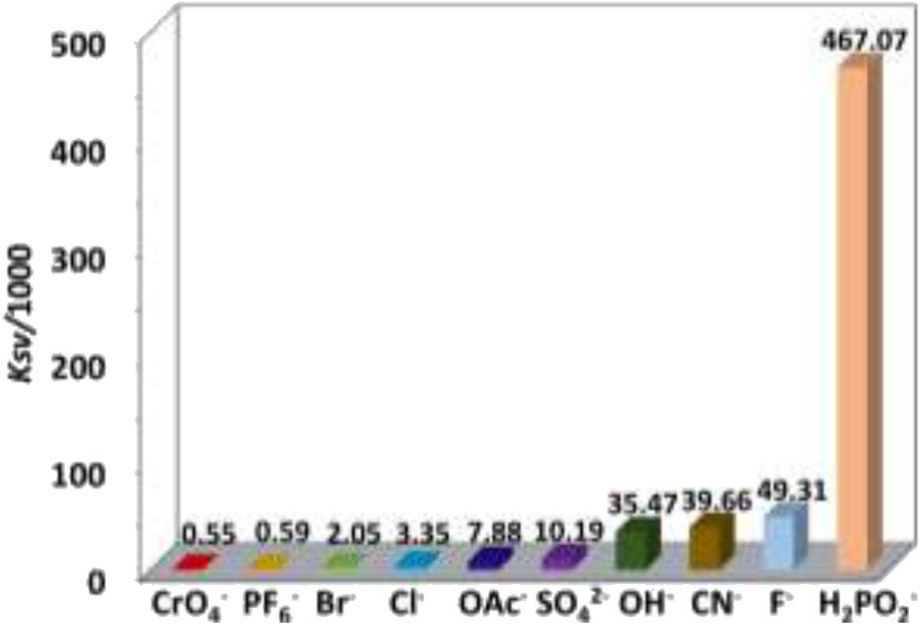
NIR emission quenching efficiencies of **1** to anions.

**Figure 7:**
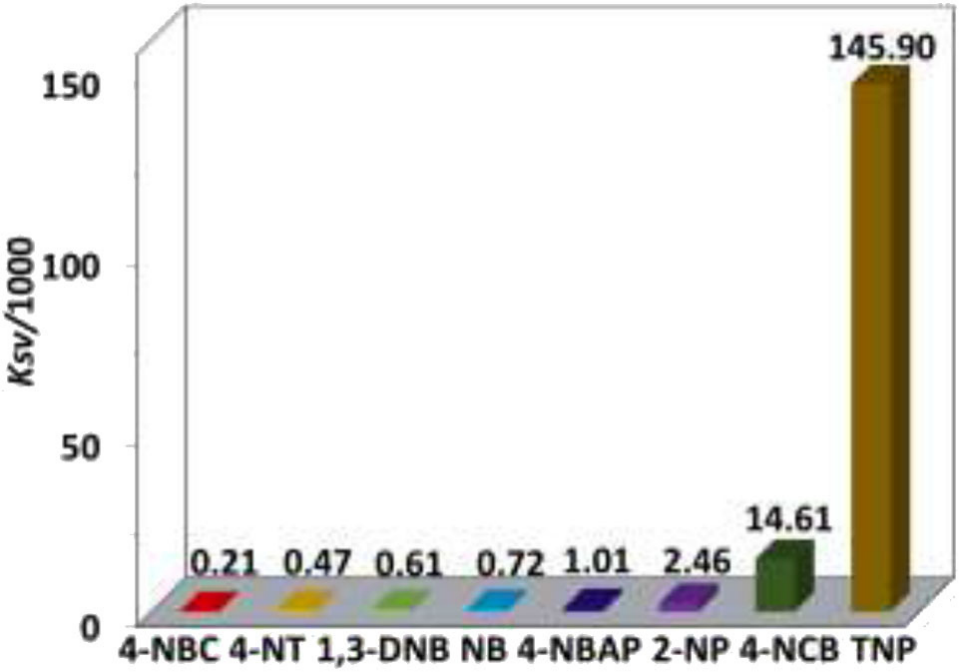
NIR emission quenching efficiencies of **1** to explosives.

The addition of anions and explosives with low concentrations, such as <5 μM for H_2_${\text{PO}}_{2^{-}}$ and TNP, results in a linear luminescence quenching response of **1**. Thus, the luminescence quenching efficiencies (*K*_*SV*_) of the complex to these analytes can be calculated using Stern–Volmer (S-V) equation *K*_SV_ = (*I*_0_/*I* – 1)/[A] (Xiao et al., 2010). As shown in Figures 6, 7, the *K*_SV_ values of **1** to H_2_${\text{PO}}_{2^{-}}$ and TNP are 4.67 × 10^5^ M^−1^ and 1.46 × 10^5^ M^−1^, respectively, which are much higher than other anions and explosives. The S–V plots of **1** can be explained by static and dynamic quenching models. The static luminescence quenching is generally associated with the formation of ground-state molecular associations upon addition of the analytes, whereas the dynamic luminescence quenching is under diffusion control, where collisions between analytes and excited fluorophores result in deactivation of the excited states. Basically, the formation of molecular associations in the static quenching model cannot affect the emission lifetimes of fluorophores, however, the collisions in dynamic luminescence quenching may reduce the emission lifetimes. The luminescence lifetimes of **1** are reduced to 4.1 and 5.2 μs with the addition of 1.8 μM of H_2_${\text{PO}}_{2^{-}}$ and 5.6 μM of TNP, respectively, indicating the dominance of dynamic luminescence quenching in **1**.

**Figure 8:**
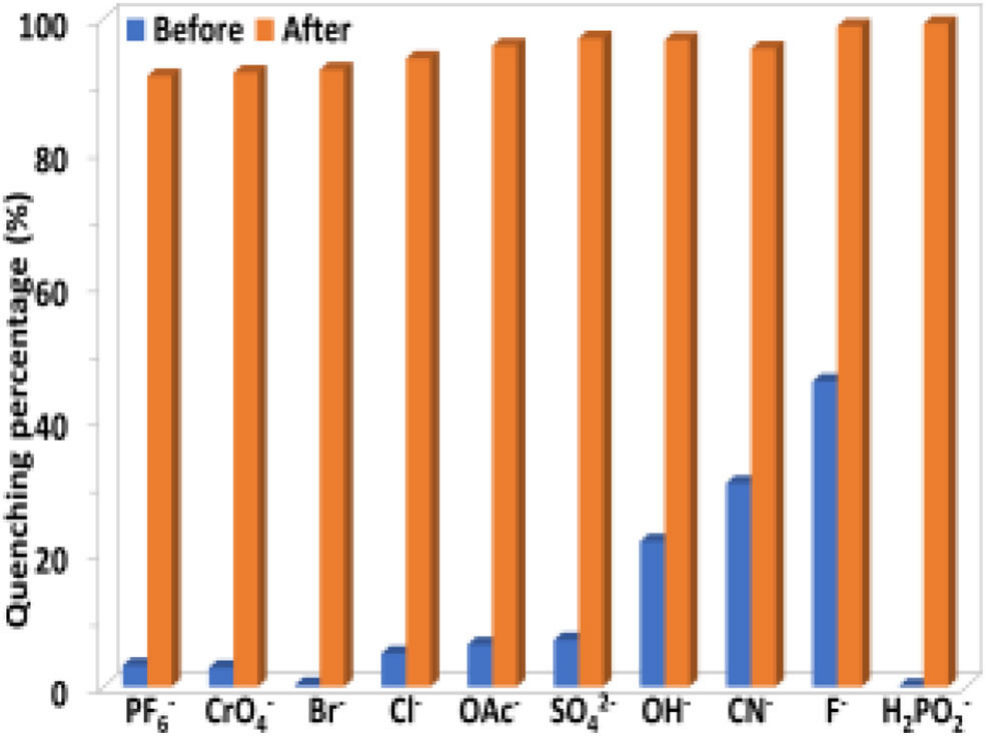
The luminescence quenching percentages of **1** (0.5 μM) before and after the addition of H_2_${\text{PO}}_{2^{-}}$ (5 μM) in the presence of other anions (5 μM) in CH_3_CN.

**Figure 9:**
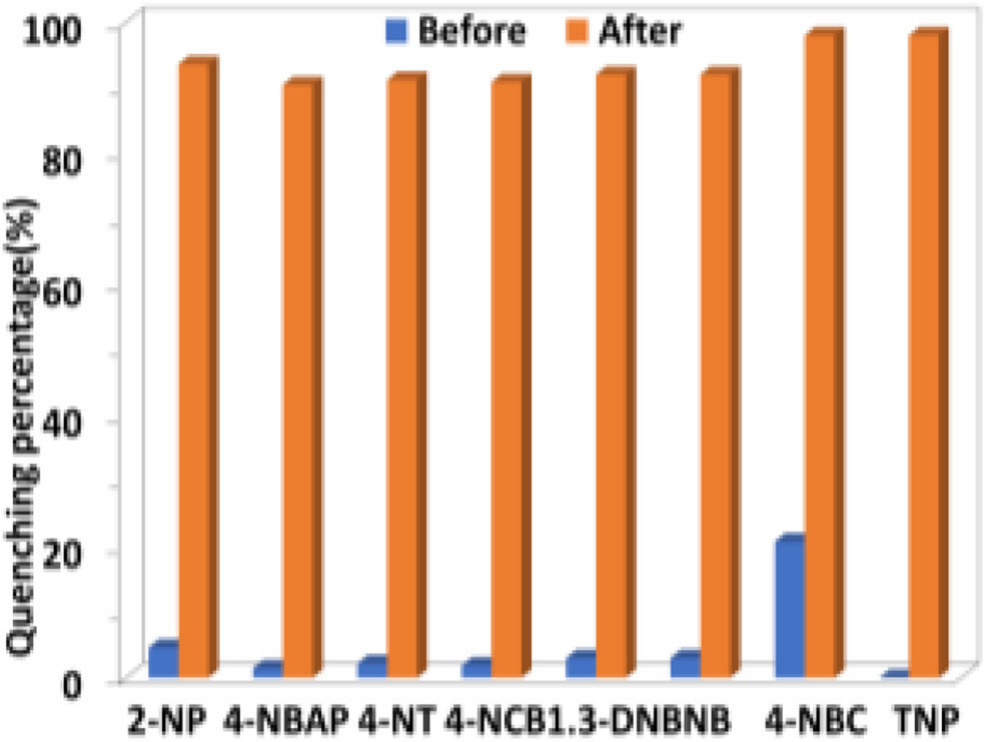
The luminescence quenching percentages of **1** (0.5 μM) before and after the addition of TNP (10 μM) in the presence of other explosives (10 μM) in CH_3_CN.

The selectivity of **1** to H_2_${\text{PO}}_{2^{-}}$ and TNP in the presence of other anions and explosives was investigated. As shown in Figures 8, 9, the existence of another anion and explosive with the same concentration does not affect the high quenching percentage of **1** to H_2_${\text{PO}}_{2^{-}}$ and TNP. These results indicate that **1** shows high selectivity to H_2_${\text{PO}}_{4^{-}}$ and TNP even in the presence of other anions and explosives, respectively.

**Figure 10:**
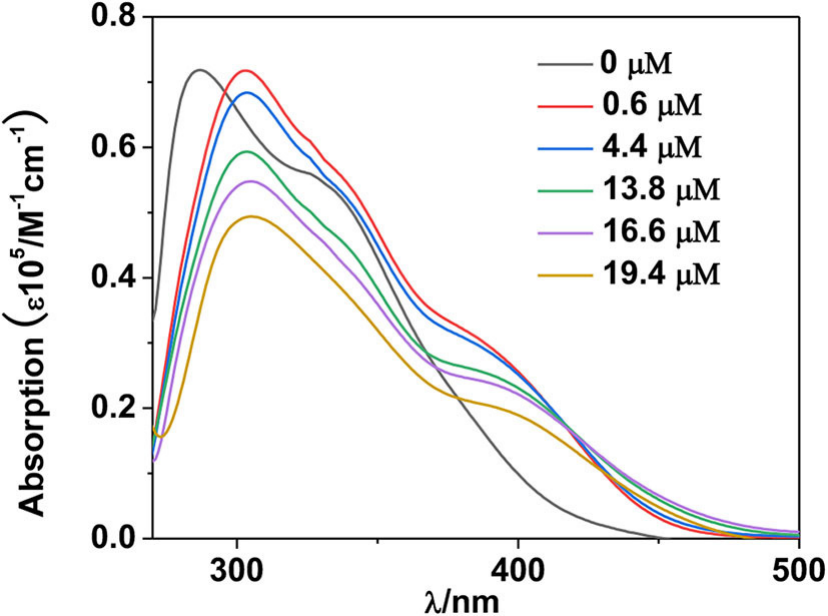
UV-visible spectra of **1** with the addition of H_2_${\text{PO}}_{2^{-}}$ anion at 298 K.

For luminescent lanthanide complexes, the LMET plays a key role in the intensities of luminescence. The electronic structure and excited state of the Schiff base ligand may be disturbed by the added anions, resulting in the change of the LMET process in **1** (Parker et al., 1998; Parker, 2000). In addition, the possible intermolecular electron transfer from anions to Schiff base ligands may also consume the excitation energy of lanthanide ions and in turn decreases the luminescence of **1** (Guha and Saha, 2010). The intermolecular interaction between the added anions and **1** is crucial to the lanthanide luminescent response. The interaction between H_2_${\text{PO}}_{2^{-}}$ anion and **1** was studied by UV-vis spectral titration (Liu et al., 2017). The red-shift of the absorption bands of **1** indicates the formation of interaction between the added H_2_${\text{PO}}_{2^{-}}$ anion and **1** (Figure 10). It is noticeable that the addition of H_2_${\text{PO}}_{2^{-}}$ anion decreases the absorption of **1** at the excitation wavelength (λ_ex_ = 395 nm), which is not advantageous for the Schiff base ligand to absorb light energy and further decreases the lanthanide luminescence (Feng et al., 2019).

**Scheme 2:**
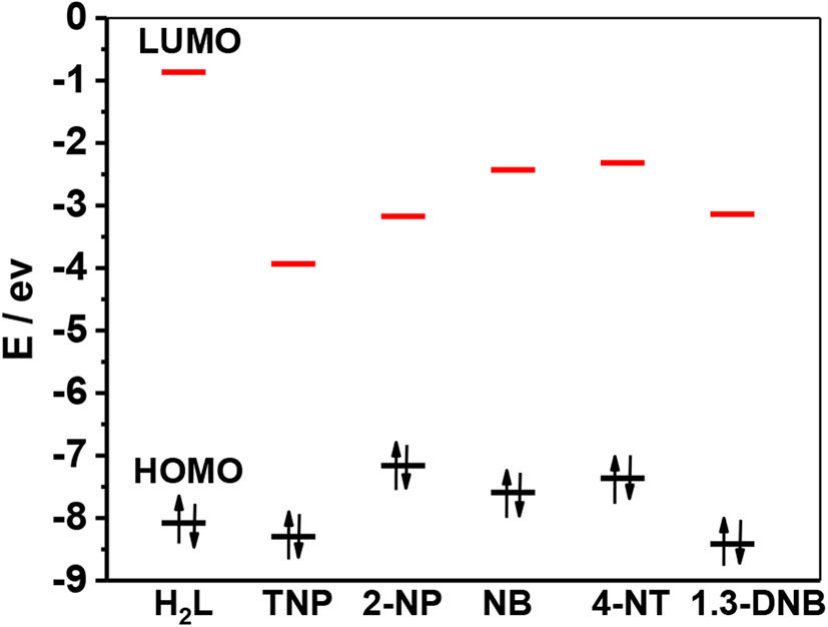
HOMO and LUMO energy levels for the free H_2_L and selected explosives.

**Figure 11:**
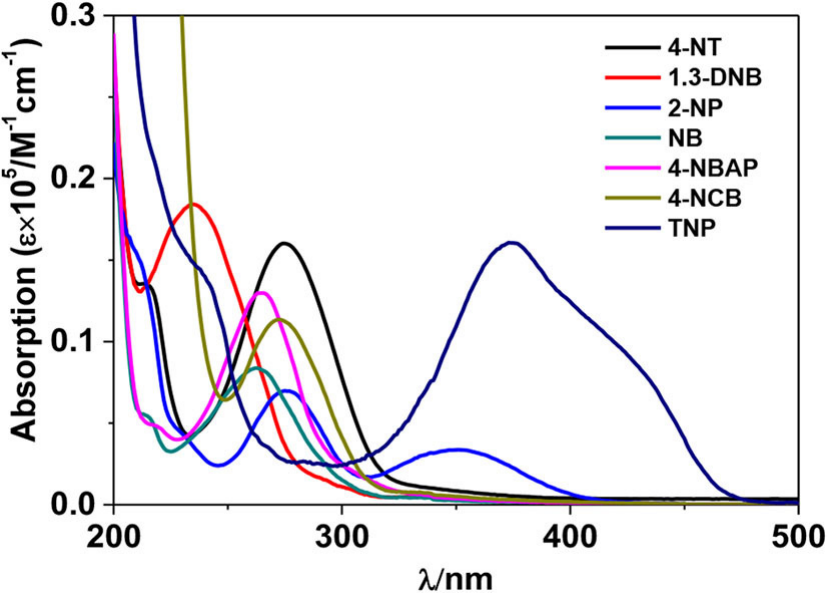
UV-vis absorption spectra of explosives in CH_3_CN.

Usually, the luminescence quenching of lanthanide complexes arisen by the addition of nitro explosives can be explained by photoinduced electron transfer mechanism (Li et al., 2013; Nagarkar et al., 2013; Qin et al., 2015). According to the literature (Xie et al., 2016), the approximate LUMO energy level of H_2_L in **1** is shown in Scheme 2, which is higher than those of explosives. Thus, the excited electrons of the Schiff base ligand can transfer to the LUMO orbitals of the explosives. TNP has the lowest LUMO energy level among the explosives, which helps in the electron transfer process. Meanwhile, the UV-vis spectra exhibit that TNP has the highest molar absorption at λ_ex_ = 395 nm, compared with other explosives (Figure 11). This indicates that TNP may compete with **1** for the excitation energy, resulting in the further decrease of lanthanide luminescence.

## Conclusions

In brief, one Zn-Nd complex **1** with dimensions of 0.8 × 1.1 × 2.8 nm was constructed from Schiff base ligand H_2_L. The structure of **1** is determined by X-ray crystallography. **1** shows the typical emission of Nd(III) under the excitation of UV-vis light. The addition of anions and nitro explosives leads to a quenching of the luminescence, with high sensitivity of **1** to H_2_${\text{PO}}_{2^{-}}$ and TNP. UV-vis spectral titration confirms the formation of interaction between H_2_${\text{PO}}_{2^{-}}$ anion and **1**, and the addition of H_2_${\text{PO}}_{2^{-}}$ anion decreases the absorption of **1** at the excitation wavelength of lanthanide luminescence. TNP has the lowest LUMO energy level among the added explosives. The addition of TNP results in the competition of excitation energy between the explosive and **1**. Further investigations focused on the construction and luminescent response properties of lanthanide-based complexes are in progress.

## Data Availability Statement

The datasets generated for this study can be found in the Cambridge Crystallographic Data Centre (CCDC number: 1971956).

## Author Contributions

XY conceived and designed the experiments. XL, YM, JL, and DS performed the experiments. MN and DS analyzed the data. All authors contributed to the article and approved the submitted version.

## Conflict of Interest

The authors declare that the research was conducted in the absence of any commercial or financial relationships that could be construed as a potential conflict of interest.
