# Supplementary material for: One Nanoscale Zn(II)-Nd(III) Complex With Schiff Base Ligand: NIR Luminescent Sensing of Anions and Nitro Explosives
Source: Front Chem. 2020 Oct 14;8:536907. doi: 10.3389/fchem.2020.536907 (PMC7591804; doi:10.3389/fchem.2020.536907)
Supplement: Supplementary file 1 [file Data_Sheet_1.doc]

# checkCIF (basic structural check) running for 1:

**Datablock: complex-1**

| Bond precision: | C-C = 0.0161 A | Wavelength=0.71073 |
| --- | --- | --- |

| Cell: | a=9.6222(19) | b=14.771(3) | c=18.303(4) |
| --- | --- | --- | --- |
|  | alpha=104.25(3) | beta=98.18(3) | gamma=103.42(3) |
| Temperature: | 190 K |  |  |

|  | Calculated | Reported |
| --- | --- | --- |
| Volume | 2397.0(11) | 2397.0(8) |
| Space group | P -1 | P-1 |
| Hall group | -P 1 | ? |
| Moiety formula | C66 H76 N4 Nd4 O32 Zn2, 2(C H6 Cl2 O2 Zn), 4(C H4 O) | ? |
| Sum formula | C72 H104 Cl4 N4 Nd4 O40 Zn4 | C72 H104 Cl4 N4 Nd4 O40 Zn4 |
| Mr | 2645.92 | 2645.83 |
| Dx,g cm-3 | 1.833 | 1.833 |
| Z | 1 | 1 |
| Mu (mm-1) | 3.305 | 3.305 |
| F000 | 1312.0 | 1312.0 |
| F000' | 1313.53 |  |
| h,k,lmax | 12,19,23 | 12,19,23 |
| Nref | 11184 | 10830 |
| Tmin,Tmax | 0.539,0.673 | 0.812,1.000 |
| Tmin' | 0.528 |  |

| Correction method= # Reported T Limits: Tmin=0.812 Tmax=1.000 AbsCorr = MULTI-SCAN |  |
| --- | --- |

| Data completeness= 0.968 | Theta(max)= 27.650 |
| --- | --- |

| R(reflections)= 0.0731( 8701) | wR2(reflections)= 0.2077( 10830) |
| --- | --- |

| S = 1.036 | Npar= 573 |
| --- | --- |

The following ALERTS were generated. Each ALERT has the format

**test-name_ALERT_alert-type_alert-level**.

Click on the hyperlinks for more details of the test.


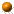
**Alert level B**

[PLAT420_ALERT_2_B](javascript:makeHelpWindow("PLAT420.html")) D-H Without Acceptor O17 --H17B . Please Check

[PLAT420_ALERT_2_B](javascript:makeHelpWindow("PLAT420.html")) D-H Without Acceptor O19 --H19D . Please Check


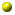
**Alert level C**

[DIFMN02_ALERT_2_C](javascript:makeHelpWindow("DIFMN_02.html")) The minimum difference density is < -0.1*ZMAX*0.75

_refine_diff_density_min given = -4.879

Test value = -4.500

[DIFMN03_ALERT_1_C](javascript:makeHelpWindow("DIFMN_03.html")) The minimum difference density is < -0.1*ZMAX*0.75

The relevant atom site should be identified.

[PLAT029_ALERT_3_C](javascript:makeHelpWindow("PLAT029.html")) _diffrn_measured_fraction_theta_full value Low . 0.968 Why?

[PLAT098_ALERT_2_C](javascript:makeHelpWindow("PLAT098.html")) Large Reported Min. (Negative) Residual Density -4.88 eA-3

[PLAT234_ALERT_4_C](javascript:makeHelpWindow("PLAT234.html")) Large Hirshfeld Difference O15 --C33 . 0.17 Ang.

[PLAT241_ALERT_2_C](javascript:makeHelpWindow("PLAT241.html")) High 'MainMol' Ueq as Compared to Neighbors of O10 Check

**And 2 other PLAT241 Alerts**

[PLAT241_ALERT_2_C](javascript:makeHelpWindow("PLAT241.html")) High 'MainMol' Ueq as Compared to Neighbors of O12 Check

[PLAT241_ALERT_2_C](javascript:makeHelpWindow("PLAT241.html")) High 'MainMol' Ueq as Compared to Neighbors of O13 Check

[PLAT242_ALERT_2_C](javascript:makeHelpWindow("PLAT242.html")) Low 'MainMol' Ueq as Compared to Neighbors of Nd2 Check

[PLAT244_ALERT_4_C](javascript:makeHelpWindow("PLAT244.html")) Low 'Solvent' Ueq as Compared to Neighbors of Zn2 Check

[PLAT260_ALERT_2_C](javascript:makeHelpWindow("PLAT260.html")) Large Average Ueq of Residue Including O18 0.192 Check

[PLAT342_ALERT_3_C](javascript:makeHelpWindow("PLAT342.html")) Low Bond Precision on C-C Bonds ............... 0.01608 Ang.

[PLAT413_ALERT_2_C](javascript:makeHelpWindow("PLAT413.html")) Short Inter XH3 .. XHn H25C ..H35A . 2.09 Ang.

1-x,2-y,1-z = 2_676 Check

[PLAT415_ALERT_2_C](javascript:makeHelpWindow("PLAT415.html")) Short Inter D-H..H-X H12A ..H17B . 2.08 Ang.

x,y,z = 1_555 Check


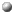
**Alert level G**

[PLAT002_ALERT_2_G](javascript:makeHelpWindow("PLAT002.html")) Number of Distance or Angle Restraints on AtSite 2 Note

[PLAT003_ALERT_2_G](javascript:makeHelpWindow("PLAT003.html")) Number of Uiso or Uij Restrained non-H Atoms ... 64 Report

[PLAT005_ALERT_5_G](javascript:makeHelpWindow("PLAT005.html")) No Embedded Refinement Details Found in the CIF Please Do !

[PLAT007_ALERT_5_G](javascript:makeHelpWindow("PLAT007.html")) Number of Unrefined Donor-H Atoms .............. 7 Report

[PLAT072_ALERT_2_G](javascript:makeHelpWindow("PLAT072.html")) SHELXL First Parameter in WGHT Unusually Large 0.11 Report

[PLAT083_ALERT_2_G](javascript:makeHelpWindow("PLAT083.html")) SHELXL Second Parameter in WGHT Unusually Large 36.02 Why ?

[PLAT152_ALERT_1_G](javascript:makeHelpWindow("PLAT152.html")) The Supplied and Calc. Volume s.u. Differ by ... 3 Units

[PLAT154_ALERT_1_G](javascript:makeHelpWindow("PLAT154.html")) The s.u.'s on the Cell Angles are Equal ..(Note) 0.03 Degree

[PLAT232_ALERT_2_G](javascript:makeHelpWindow("PLAT232.html")) Hirshfeld Test Diff (M-X) Nd2 --O14 . 5.3 s.u.

[PLAT380_ALERT_4_G](javascript:makeHelpWindow("PLAT380.html")) Incorrectly? Oriented X(sp2)-Methyl Moiety ..... C23 Check

[PLAT380_ALERT_4_G](javascript:makeHelpWindow("PLAT380.html")) Incorrectly? Oriented X(sp2)-Methyl Moiety ..... C29 Check

[PLAT432_ALERT_2_G](javascript:makeHelpWindow("PLAT432.html")) Short Inter X...Y Contact O13 ..C35 2.83 Ang.

1-x,2-y,1-z = 2_676 Check

[PLAT432_ALERT_2_G](javascript:makeHelpWindow("PLAT432.html")) Short Inter X...Y Contact O16 ..C35 2.75 Ang.

x,y,z = 1_555 Check

[PLAT774_ALERT_1_G](javascript:makeHelpWindow("PLAT774.html")) Suspect X-Y Bond in CIF: Nd1 --Nd2 .. 4.05 Ang.

[PLAT790_ALERT_4_G](javascript:makeHelpWindow("PLAT790.html")) Centre of Gravity not Within Unit Cell: Resd. # 4 Note

C H4 O

[PLAT794_ALERT_5_G](javascript:makeHelpWindow("PLAT794.html")) Tentative Bond Valency for Nd1 (II) . 2.42 Info

**And 3 other PLAT794 Alerts**

[PLAT794_ALERT_5_G](javascript:makeHelpWindow("PLAT794.html")) Tentative Bond Valency for Nd2 (II) . 2.40 Info

[PLAT794_ALERT_5_G](javascript:makeHelpWindow("PLAT794.html")) Tentative Bond Valency for Zn1 (II) . 2.17 Info

[PLAT794_ALERT_5_G](javascript:makeHelpWindow("PLAT794.html")) Tentative Bond Valency for Zn2 (II) . 1.86 Info

[PLAT860_ALERT_3_G](javascript:makeHelpWindow("PLAT860.html")) Number of Least-Squares Restraints ............. 385 Note

[PLAT899_ALERT_4_G](javascript:makeHelpWindow("PLAT899.html")) SHELXL97 is Deprecated and Succeeded by SHELXL 2018 Note

**PLATON version of 07/08/2019; check.def file version of 30/07/2019**

| **Datablock complex-1** - ellipsoid plot |
| --- |
| 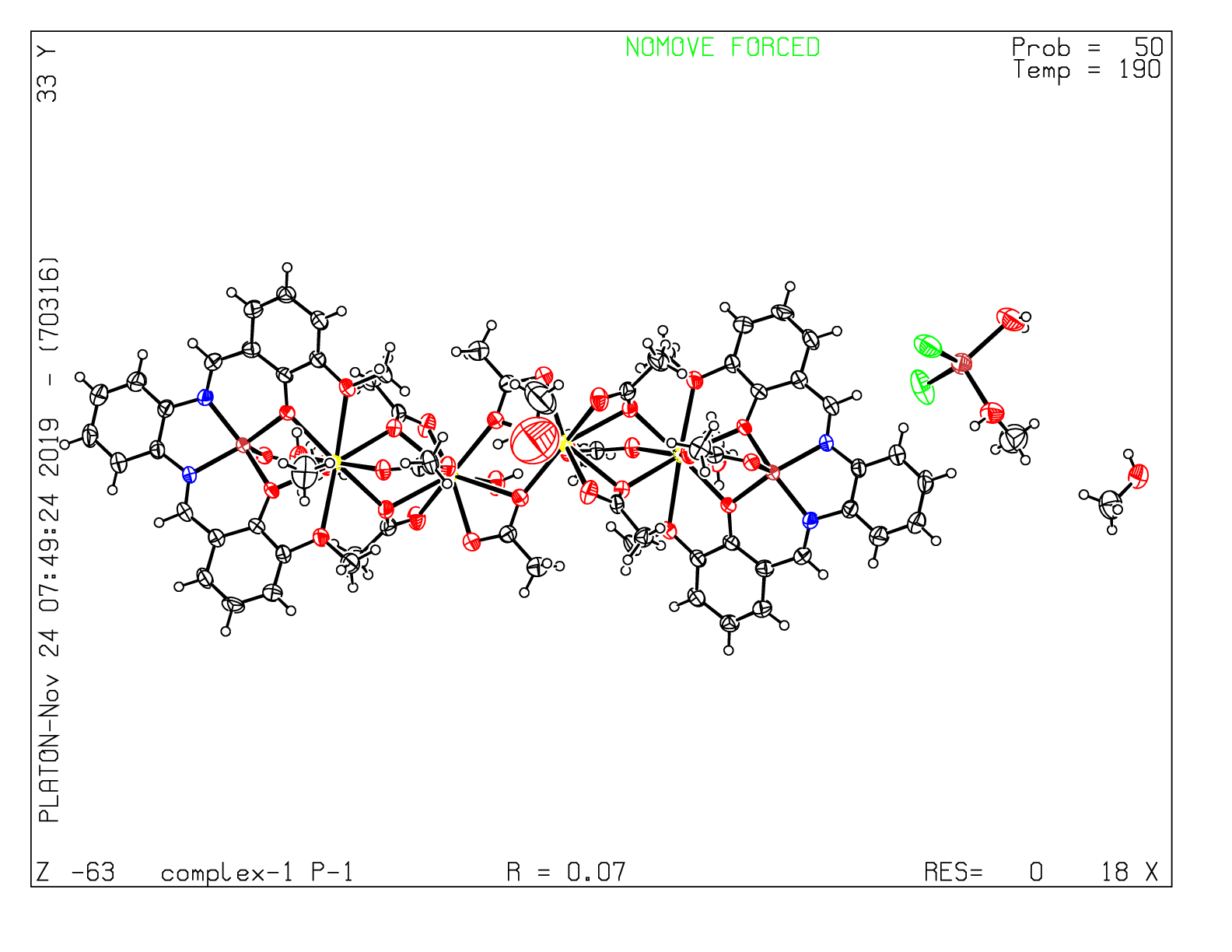 |

[Download CIF editor (publCIF) from the IUCr](http://journals.iucr.org/services/cif/publcif/)
[Download CIF editor (enCIFer) from the CCDC](http://www.ccdc.cam.ac.uk/free_services/encifer/)
[Test a new CIF entry](http://checkcif.iucr.org/index.html)
