# Supplementary material for: One Nanoscale Zn(II)-Nd(III) Complex With Schiff Base Ligand: NIR Luminescent Sensing of Anions and Nitro Explosives
Source: Front Chem. 2020 Oct 14;8:536907. doi: 10.3389/fchem.2020.536907 (PMC7591804; doi:10.3389/fchem.2020.536907)
Supplement: Supplementary file 2 [file Data_Sheet_2.docx]

**Supporting Information**

**One nanoscale Zn(II)-Nd(III) complex with Schiff base ligand: NIR luminescent sensing of anions and nitro explosives**

Xia Liu ^1^, Xiaoping Yang ^1^*, Yanan Ma ^1^, Jieni Liu ^1^, Dongliang Shi ^1^, Mengyu Niu ^1^ and Desmond Schipper ^2^

**Contents**

[1. General procedures S2](#_Toc23863763)

[2. IR spectra of free ligand H_2_L and complex **1** S3](#_Toc23863766)

[3. The mogravimetric analysis of **1** S3](#_Toc23863767)

[4. Powder XRD pattern of **1** S4](#_Toc23863768)

[5. Chemical structures of nitro explosives S5](#_Toc23863768)

[6. NIR luminescent response of **1** to anions S6](#_Toc23863772)

[7. NIR luminescent response of **1** to explosives S9](#_Toc23863772)

[8. X-Ray crystallography S11](#_Toc23863774)

**1. General procedures**

All reactions were performed under dry oxygen-free dinitrogen atmospheres using standard Schlenk techniques. The Schiff-base ligand H_2_L was prepared according to well established procedures.^1^ NMR spectra were carried out with a AVANCE IIIA V500 spectrometer at 298 K using CDCl_3_ as the solvent; IR spectra were recorded at 298 K on a Nicolet IS10 spectrometer, equipped with a UATR sampling accessory. Powder XRD measurements were performed in a D8ADVANCE diffractometer using Cu Kα radiation, 40 kV, 40 mA, at a scan rate of 0.02 (in 2θ / degree) and a step of 10 s per point. Elemental analyses (C, H, N) of compounds were carried out on a EURO EA3000 elemental analysis after dried in an oven at 100 ^o^C for 2 h. Melting points were obtained in sealed glass capillaries under dinitrogen and are uncorrected. The thermogravimetric analyses (TA) were carried out on a TA Instrum -ents Q600 at a heating rate of 2 ^o^C min^-1^ from room temperature to 800 ^o^C. Conductivity measurement was carried out with a DDS-11 conductivity bridge for 10^-4^ M solution in CH_3_CN. Absorption spectra were obtained on a UV-3600 spectrophotometer in the range of 200-600 nm. Field emission scanning electron microscopy (FESEM) images were recorded on a Nova NanoSEM 200 scanning electron microscope.

**Photophysical Studies** NIR luminescence spectra were recorded on a FLS 980 fluorimeter. The light source for the spectra was a 450 W xenon arc lamp with continuous spectral distribution from 190 to 2600 nm. Liquid nitrogen cooled Ge PIN diode detector was used to detect the NIR emissions from 800 nm to 1700 nm. The temporal decay curves of the fluorescence signals were stored by using the attached storage digital oscilloscope. The quantum yields (Φ_em_) were obtained by using an integrating sphere, according to eqn Φ_em_ = *N*_em_ / *N*_abs_, where *N*_em_ and *N*_abs_ are the numbers of emitted and absorbed photons, respectively. Systematic errors have been deducted through the standard instrument corrections. All the measurements were carried out at room temperature.

Ref. 1. Lam, F.; Xu, J.-X.; Chan, K.-S. *J. Org. Chem.*, **1996**, *61*, 8414-8418.

**2. IR spectra of free ligand H_2_L and complex 1**

**Figure S1**. IR spectra of the free ligand H_2_L and complex **1**.

**3. The thermogravimetric analysis of 1**

**Figure S2**. The thermogravimetric analysis of **1**.

**4. Powder XRD pattern of 1**


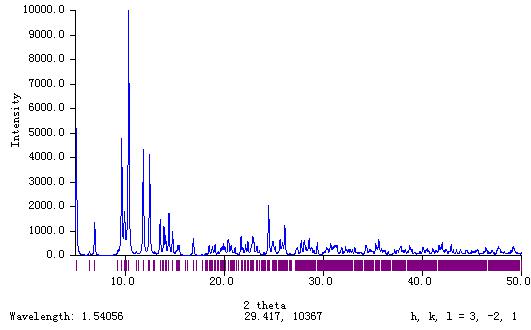

**Figure S3**. Powder XRD pattern of **1**.

**5. Chemical structures of nitro explosives**

**Scheme S1.** Chemical structures of nitro explosives.

**6. NIR luminescent response of 1 to anions**

**Figure S4.** Luminescent sensing of **1** (5 μM) to anions in CH_3_CN (λ_ex_ = 395 nm).

**7. NIR luminescent response of 1 to explosives**

**Figure S5.** Luminescent sensing of **1** (5 μM) to explosives in CH_3_CN (λ_ex_ = 395 nm).

**8. X-Ray crystallography**

**Table S1**. Selected Bond Lengths (Å) and Angles (°) for **1**.

Nd(1)-O(7) 2.314(6)

Nd(1)-O(3) 2.366(6)

Nd(1)-O(2) 2.367(6)

Nd(1)-O(5) 2.373(7)

Nd(1)-O(15) 2.413(7)

Nd(1)-O(11) 2.454(7)

Nd(1)-O(9) 2.464(6)

Nd(1)-O(4) 2.648(7)

Nd(1)-O(1) 2.679(7)

Nd(2)-O(16) 2.372(8)

Nd(2)-O(8) 2.397(7)

Nd(2)-O(12) 2.425(7)

Nd(2)-O(13) 2.435(8)

Nd(2)-O(9) 2.464(6)

Nd(2)-O(10) 2.465(7)

Nd(2)-O(11) 2.488(7)

Nd(2)-O(14) 2.581(7)

Zn(1)-O(6) 1.994(7)

Zn(1)-O(3) 2.003(7)

Zn(1)-O(2) 2.012(6)

Zn(1)-N(1) 2.036(8)

Zn(1)-N(2) 2.047(7)

O(7)-Nd(1)-O(3) 133.5(2)

O(7)-Nd(1)-O(2) 132.9(2)

O(3)-Nd(1)-O(2) 67.8(2)

O(7)-Nd(1)-O(5) 143.6(2)

O(3)-Nd(1)-O(5) 73.4(2)

O(2)-Nd(1)-O(5) 74.9(2)

O(7)-Nd(1)-O(15) 74.9(2)

O(3)-Nd(1)-O(15) 72.3(2)

O(2)-Nd(1)-O(15) 75.9(2)

O(5)-Nd(1)-O(15) 141.3(2)

O(7)-Nd(1)-O(11) 74.8(2)

O(3)-Nd(1)-O(11) 108.0(2)

O(2)-Nd(1)-O(11) 147.2(2)

O(5)-Nd(1)-O(11) 72.9(2)

O(15)-Nd(1)-O(11) 135.4(2)

O(7)-Nd(1)-O(9) 79.3(2)

O(3)-Nd(1)-O(9) 146.0(2)

O(2)-Nd(1)-O(9) 97.5(2)

O(5)-Nd(1)-O(9) 73.2(2)

O(15)-Nd(1)-O(9) 135.9(2)

O(11)-Nd(1)-O(9) 67.3(2)

O(7)-Nd(1)-O(4) 80.0(2)

O(3)-Nd(1)-O(4) 60.3(2)

O(2)-Nd(1)-O(4) 125.9(2)

O(5)-Nd(1)-O(4) 102.9(2)

O(15)-Nd(1)-O(4) 74.9(3)

O(11)-Nd(1)-O(4) 68.2(2)

O(9)-Nd(1)-O(4) 134.4(2)

O(7)-Nd(1)-O(1) 76.2(2)

O(3)-Nd(1)-O(1) 122.9(2)

O(2)-Nd(1)-O(1) 60.4(2)

O(5)-Nd(1)-O(1) 112.2(2)

O(15)-Nd(1)-O(1) 73.3(2)

O(11)-Nd(1)-O(1) 128.5(2)

O(9)-Nd(1)-O(1) 66.0(2)

O(4)-Nd(1)-O(1) 144.2(2)

O(16)-Nd(2)-O(8) 143.8(2)

O(16)-Nd(2)-O(12) 74.6(3)

O(8)-Nd(2)-O(12) 132.7(3)

O(16)-Nd(2)-O(13) 85.3(3)

O(8)-Nd(2)-O(13) 81.2(3)

O(12)-Nd(2)-O(13) 75.7(3)

O(16)-Nd(2)-O(9) 129.6(3)

O(8)-Nd(2)-O(9) 78.2(2)

O(12)-Nd(2)-O(9) 94.2(2)

O(13)-Nd(2)-O(9) 140.3(2)

O(16)-Nd(2)-O(10) 76.7(3)

O(8)-Nd(2)-O(10) 125.5(3)

O(12)-Nd(2)-O(10) 79.1(3)

O(13)-Nd(2)-O(10) 152.1(3)

O(9)-Nd(2)-O(10) 52.9(2)

O(16)-Nd(2)-O(11) 126.9(3)

O(8)-Nd(2)-O(11) 82.4(2)

O(12)-Nd(2)-O(11) 52.7(2)

O(13)-Nd(2)-O(11) 77.1(2)

O(9)-Nd(2)-O(11) 66.8(2)

O(10)-Nd(2)-O(11) 96.8(2)

O(16)-Nd(2)-O(14) 70.5(2)

O(8)-Nd(2)-O(14) 74.7(2)

O(12)-Nd(2)-O(14) 117.7(2)

O(13)-Nd(2)-O(14) 51.8(2)

O(9)-Nd(2)-O(14) 147.3(2)

O(10)-Nd(2)-O(14) 136.1(2)

O(11)-Nd(2)-O(14) 126.1(2)

O(6)-Zn(1)-O(3) 97.6(3)

O(6)-Zn(1)-O(2) 102.8(3)

O(3)-Zn(1)-O(2) 82.2(3)

O(6)-Zn(1)-N(1) 112.0(3)

O(3)-Zn(1)-N(1) 150.4(3)

O(2)-Zn(1)-N(1) 91.1(3)

O(6)-Zn(1)-N(2) 106.6(3)

O(3)-Zn(1)-N(2) 91.2(3)

O(2)-Zn(1)-N(2) 150.5(3)

N(1)-Zn(1)-N(2) 80.5(3)
